# Effect of Shape, Size, and Color of the Food Plate on Consumer Perception of Energy Value, Portion Size, Attractiveness, and Expected Price of Dessert

**DOI:** 10.3390/foods13132063

**Published:** 2024-06-28

**Authors:** Artur Głuchowski, Katarzyna Koteluk, Ewa Czarniecka-Skubina

**Affiliations:** Department of Food Gastronomy and Food Hygiene, Institute of Human Nutrition Sciences, Warsaw University of Life Sciences (WULS), Str. Nowoursynowska 166, 02-787 Warsaw, Poland; artur_gluchowski@sggw.edu.pl (A.G.);

**Keywords:** appetite, sensory properties, hedonics, portion size, plate color and shape, acceptability, energy value, expected price

## Abstract

The development of new dishes in the catering services market requires an understanding of consumers’ needs, expectations, and motivations for their choices. The effect of the serving method of a dessert on customers’ perceptions of its visual appeal, portion size, energy value, and expected price was evaluated. The study involved the presentation of desserts on plates of various sizes, shapes, and colors. The study was carried out among 1005 respondents using the CAWI method. Our findings revealed that along with an increasing plate size from a diameter of ϕ24–27 cm to ϕ31 cm, the ratings of the dish’s perceived appearance (*p* ≤ 0.001), portion size (*p* ≤ 0.001), and energy value (*p* ≤ 0.01) decreased. Plate shape influenced the perceived appearance of the dessert. When placed on a square platter, round desserts were considerably (*p* ≤ 0.05) less appealing. The color of the plate had a significant influence (*p* ≤ 0.001) on the dish’s perceived appearance and estimated monetary value, and it evoked more sensory–hedonic impressions. Red-plate and white-plate desserts were liked less than black-plate desserts, but color-plated desserts were perceived as more expensive than those served on white dishes. Consumers perceived bright desserts on white plates as traditional, natural, and boring; those on black plates as modern, appetizing, and aesthetic; and those served on red plates as artificial, unsightly, and unappetizing. Higher consumer food neophobia led to a lower rating related to appearance and price perceptions, but elevated perceptions of portion size and energy value appraisal. Our results may be used in the marketing of gastronomic dishes.

## 1. Introduction

Eating behavior refers to a complex interplay that influences motivation, meal choices, preferences, and food intake in response to environmental cues [1,2]. The dining experience is a multifactorial and dynamic phenomenon. Food perception and acceptance are driven by many intrinsic and extrinsic food-related factors [3]. The scientific literature has investigated environmental factors (visual, auditory, olfactory, and touch cues) that significantly influence consumer perception and behavior, and this has been thoroughly reviewed by Spence and Piqueras-Fiszman [4] and Seo [5]. There are numerous visual characteristics that contribute to making food appear more appetizing, including symmetry, shape, freshness, glossiness, and dynamic presentation [6]. High-end restaurants provide a variety of stimuli that affect customers’ perceptions of chefs’ signature dishes, especially the plate design: its’ material, size, shape, and color [7]. They attempt to attract customers, who increasingly demand new flavors, pleasure, and fun [8].

Visual cues are an essential part of the overall eating experience and have long been important for people. Food can be intentionally discolored to surprise or confuse, signal flavor, or capture the consumer’s attention. The significant impact of background color, like plateware, glassware, or food packaging, is also highlighted [9]. Graphic representations of appealing foods can influence our eating behavior by evoking food cravings and redirecting food selection toward unhealthy options [10]. Color contrast between plateware and food can create people’s flavor expectations, making the latter appear more vivid and, therefore possibly more appealing [11]. Individuals associate taste qualities with product design features like curvature, symmetry, orientation, and texture [12].

Many studies on food color [3,13], receptacle color [14,15,16,17], vessel shape [14,15,16,17,18,19], food plate size [19,20,21,22,23,24], food arrangement [25,26,27], and the projection-based dynamic texture of food [28] have been carried out. On the other hand, only a few studies focus on the effect of the serving manner on portion size, expected price, and caloric value [29]. Additionally, to the best of our knowledge, none have assessed multiple features in a large sample of consumers.

Although manipulating food containers seems to be an effective and simple approach to controlling satiety [22], the literature on whether changing plate size affects consumption is varied and contradictory [30]. Neurocognitive and implicit measures based on food images have been particularly effective in the investigation of subtle appetitive and regulatory factors of overt eating behavior [10]. The average perceived caloric content corresponds accordingly to the actual caloric content. Food images may evoke a reflexive emotional reaction, followed by an evaluative stage [31].

For these reasons, the study aimed to assess the effect of the serving manner of a vanilla–chocolate dessert on consumers’ perceptions of its appearance, portion size, price, and energy value. The study explores the effects of the size, shape, and color of the plate. In addition, the influence of food neophobia and consumer innovation, as well as socio-demographic factors, are taken into account.

The following hypotheses were made:

- The color, size, and shape of the plate will affect the consumer’s perception of dessert.
- Colorful plates and plates other than round (standard) plates will improve dish liking.
- Food neophobia will have a significant effect on dessert served on different plates.

## 2. Materials and Methods

### 2.1. Study Design

**Figure 1:**
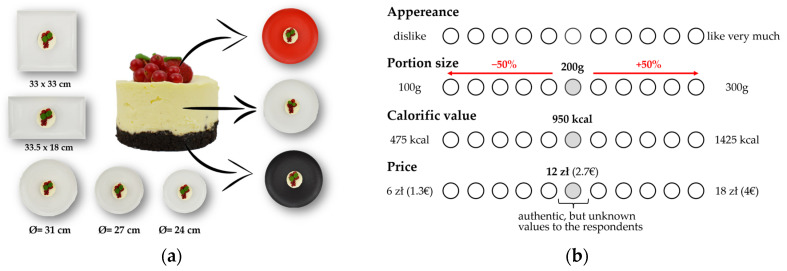
(**a**) Study design visualization. (**b**) Explanation of the idea of the scale concept.

The study had a multifactorial design (Figure 1) and evaluated the consumer’s perception of energy value, willingness to buy, and attractiveness of the same dessert served on plates of various sizes (Ø = 24, 27, 31cm), shapes (round, rectangular, and square), and colors (white, red, and black).

### 2.2. Study Material 

The study material was vanilla mascarpone cream served on a chocolate cookie crust with red berries and mint on top. The dessert consisted of mascarpone cheese, whipped cream, vanilla sugar, butter, crushed chocolate cookie pieces, raspberry, and redcurrant. The dessert portion (visual stimuli) presented on photographs weighs approximately 200 g and contains 950 kcal. The photos of the dessert were taken by Artur Głuchowski. The photo presentation was randomized to avoid biases.

### 2.3. Data Collection

The Computer-Assisted Web-based interviewing (CAWI) method using the Google Forms platform (Google Poland sp. z o.o., Warsaw, Poland) was used to collect all data. The survey was conducted in a group of 1005 adult respondents in Poland between November 2021 and April 2022. 

A link to the questionnaire in Polish was sent to respondents via social media, e-mail, and instant messaging channels. Forms were completed anonymously and at a convenient time for consumers. 

The following were the survey’s inclusion criteria:

1. Polish-speaking adult subjects, who gave consent to participate in the study.
2. Subjects who declared no visual impairments.

This paper was designed as a study with convenience sampling.

The proposed questionnaire was checked during a pilot study involving five people. All inaccuracies were identified, and the questionnaire was revised. Completing the proposed form by participants took approximately 15–20 min. 

### 2.4. Questionnaire

The questionnaire consisted of three parts (Table S1). The first concerned the frequency of the use of catering services and the importance of factors influencing perceptions of dishes (7 questions in total). Additionally, the degree of food neophobia and consumer innovation were assessed. For this purpose, the respondents indicated on a 7-point Likert scale (1—strongly agree and 7—strongly disagree) to what extent they agree with 16 statements. The first ten items assessed the food neophobia level according to the Food Neophobia scale (FNS) [32] adapted for the purposes of this study. The remaining six items evaluated domain-specific innovativeness [33].

In the main part of the study, respondents were asked to carefully look at the photo displayed on the screen and then, on a scale, to assess the elements they deemed important when choosing a dish in a catering establishment (Figure 1b):

- appearance (dislike–like very much);
- portion size (100–300 g);
- price (PLN 6–18 = EUR 1.3–4);
- energy value (475–1425 kcal).

The collage of photos used in this study is presented in the supplemental data (Figure S2). The dessert cutlery was placed above the plate as a reference point, and the side view of the dessert was shown before the assessment. Consumers assessed the elements using an 11-point structured graphic scale. The anchors on the edges were ±50% of the actual weight, price, and energy value. 

The subjects were additionally asked to check as many sensory and hedonic impressions as they wished when looking at photographs of dishes served on colorful plates. For this purpose, CATA (check-all-that-apply), a multiple-choice consumer-based method, was used. Attributes were selected by the assessors and a panel leader in the pilot study. The sensory descriptors were elicited by presenting photographs of a dessert to a group of assessors, who freely stated sensory attributes that were later gathered and selected by the Panel Leader as the most frequent ones. Participants were asked to focus on the overall sensory impression of the displayed photos and tick all associated hedonic and emotional expressions (surprising, boring, traditional, modern, appetizing, unappetizing, cheap, expensive, aesthetic, unsightly, natural, and artificial). 

The last part of the questionnaire concerned the socio-demographic details of the respondent (gender, age, place of dwelling, and financial situation).

### 2.5. Characteristics of Respondents

The study primarily involved adult women (76.6%), younger than 25 years old (66.4%), and people residing mainly in cities (70.8%), as shown in Table 1. Participants had mostly secondary (47.5%) or higher education (37.6%) and declared their financial situation as good (53.2%) or very good (15.0%).

The actual Food Neophobia scores ranged from 10 to 69 (max. 70), wherein higher values correspond to a greater neophobia level. The surveyed consumers were divided into three groups: the most neophiliac (10.0–21.4), neutral (21.4–41.8), and the most neophobic subjects (41.8–70.0). Cutoff points were calculated by adding or subtracting one standard deviation (10.2) from the mean value (31.6). This classification has been applied in many studies, as reported by Vidigal et al. [34]. The domain-specific innovativeness scores were between 6 and 42. The mean value in the group was 23.9 (SD ± 6.6). Utilizing the 33rd and 66th percentile points as cutoffs, consumers were classified into three groups: adapters (6–21), neutrals (22–26), and innovators (27–42). 

The group of respondents (Table 1) was half neophiliac (47.7%) and half neutral (48.0%). Only a minority (4.4%) was classified as neophobic.

### 2.6. Statistical Analysis 

The analysis of statistical data was performed using Statistica^®^ 13.3 (TIBCO Software Inc., Palo Alto, CA, USA). The Shapiro–Wilk test was used to verify the normality of the data distribution. The level of materiality at which the results were deemed statistically significant was equal to 0.05.

To compare the differences in perceived appearance, perceived portion size, expected price, and perceived energy value depending on plate type and socio-demographic features, the Kruskal–Wallis test with the Dunn post-hoc test were performed.

Spearman’s rank correlation coefficients were calculated to relate the perception of energy value and portion size, as well as the given hedonic-emotional impressions from the CATA analysis.

A correspondence analysis based on chi-square distance was used to visualize relationships between the sensory and hedonic impressions indicated by consumers in the CATA method and the plate colors. Cochrane’s Q test was used to find if the proportion of customers who chose each attribute of the CATA question was altered when the plate colors were taken into consideration. McNemar’s test with the Bonferroni alpha correction was used for post-hoc multiple pairwise comparisons.

## 3. Results and Discussion

### 3.1. Effect of Plate Size on Dessert Perception

There was a significant effect of plate size on dish perception among consumers. Along with the increased size of the round plate (from a diameter of ϕ24–27 cm to ϕ31 cm), the perceived appearance of the dish (*p* = 0.000), as well as the perceived portion size (*p* = 0.000) and the perceived energy value (*p* = 0.015), had decreased. As expected, the estimation of energy value was related to plate size (r = 0.63, *p* ≤ 0.05). Interestingly, the portion size and energy value of the dessert served on the biggest plate were the closest to the actual one (Table 2).

Dessert served on a matte 31-diameter white plate (Section 3.3, Table 4) was perceived by consumers as significantly (0 ≤ 0.05) more appealing (+3.0%), bigger (+4.2%), and more calorific (+3.2%) than one served on the glossy variation (Table 2).

Our findings result from the Delboeuf optico-geometric illusion, wherein outer rings make the central disk appear bigger than it actually is [4,35]. A phenomenon describing how portion size can substantially influence food intake is known as the portion-size effect [36].

In this study, dessert was arranged vertically and participants were acquainted with a side view. Rowley and Spence [23] found that the plate of food was rated as having a bigger portion size when the elements were arrayed horizontally rather than stacked vertically. Moreover, the centrally-plated dessert seems to have a larger portion size, when compared to the offset version of the same dish. This effect may stem from the consumer tendency to base portion size assessments on the length and width dimensions of the food as these dimensions would be relatively more salient than the height dimension [37]. This type of visual manipulation is more easily facilitated in laboratory-based tasting studies. Nevertheless, in free-living settings, portion size is often partially predetermined by the container size in which food is sold [24]. In our study, dessert cutlery was used as a reference point on every displayed photograph of a food plate. Nevertheless, consumers probably focus their attention on the food plate surface and thin rims. As demonstrated by McClain et al. [20] participants overestimated the diameter of food portions by 5% and the visual area of food portions by 10% on plates with wider rims compared to those with very thin rims. Congruent results are also presented by Huang et al. [38], who suggested that restaurants could offer dishes on bigger platters to reduce food waste by establishing perceived scarcity.

Opposite conclusions were drawn by Penaforte et al. [19], who concluded that the size of the plate has no effect on food portion estimates, even if it did affect the classification of portion size. A meta-analysis performed by Holden et al. [30] showed that plate size significantly affects the amount of food that is served and consumed when portion size is self-served or manipulated, but not when portion size is constant. Conclusions were drawn that plate size has a stronger effect when participants are unaware of the experiment. The success of plate-size effects is also associated with the susceptibility of individuals. The study of Peng [39] revealed differences in susceptibility between normal-weight and overweight groups, while Sim and Cheon [40] examined these differences between individuals with a dispositional tendency to process information in a holistic (vs. analytic) manner. The plate-size effect was more powerful for men than women [41] and differed between Asian and Western respondents [42]. Small tableware may increase post-meal satiety and initially reduce energy intake. However, subsequent meals may equalize energy intake, suggesting that this may not be a long-term solution for combating overconsumption [43]. Moreover, portion size can have a greater impact on meal planning than actual food intake [36]. 

### 3.2. Effect of Plate Shape on Dessert Perception

Plate shape only affected the appearance of the dessert. Round-shaped desserts served on round and rectangular plates were significantly (*p* ≤ 0.05) more appealing (+2.6–6.7%) than when served on a square plate (Table 3). No significant effect was seen in the perception of portion size, energy value, and menu price. The higher variations in the assessment of non-round plates indicate that the use of unusual shapes has both supporters and opponents, while the round option was perceived as the standard one. 

Consumers generally prefer curved (vs. angular) objects, as evidenced in research on chocolate, water, cars, pills, and cookie packaging [44]. However, the results of Piqueras-Fiszman et al. [17] demonstrated that the shape of the plate has an insignificant effect on people’s perception of the food. Steward and Goss [15] also did not find any notable effects of plate shape on liking ratings. There was a significant influence of shape in the case of black plates, but not white ones. These results suggest that plate shape and color may affect taste perception, though not in a straightforward manner. This more complex influence depends on the interaction of the two variables. The complexity was also confirmed in the findings of Jang et al. [44], who found that round (vs. square) plates elicited more favorable attitudes toward the restaurant only when presented in an organized manner. A less organized manner of the presentation sides blurred the plate shape effect. Liked tastes are paired with preferred shapes, while disliked tastes are associated with threatening shapes. Studies suggest a semantic differential space with hedonics and intensity can account for people’s responses to taste [12].

### 3.3. Effect of Plate Color on Dessert Perception

The plate color had a significant effect on the dish’s perceived appearance (*p* = 0.000) and expected menu price (*p* = 0.000), Table 4. 

The color did not affect the portion size and energy value perception of the surveyed consumers. Dessert served on a red plate (6.8 c.u.) and on a white one (7.1 c.u.) was liked less than the dessert served on a black plate (8.0 c.u.). Dessert served on colorful plates (14.22–14.72 PLN) was assessed as significantly more expensive (+2.1–5.7%) than dessert served on white plates (13.93 PLN), and dessert served on black plates was considered the most expensive.

The results of Piqueras-Fiszman et al. [17] revealed that plate color had a significant influence on people’s perception of the mousse. Pink mousse served on a white plate was perceived by consumers not only as more liked but also as significantly more intense and sweeter. Moreover, in the research of Stewart and Goss [15], cheesecake was also affected by plate color. Sweetness or flavor intensity was enhanced by white round plates, while quality or liking was enhanced by both white round and black square plates. Moreover, treacle tart with ice cream was more liked by a group of consumers who had dessert served on a square black plate than the group consuming from a round white plate [45]. Food plate color can affect people’s perceptions of traits based on a visual appraisal. Nevertheless, the effect cannot be explained by color contrast alone. Other results by Piqueras-Fiszman et al. [16] revealed that the contrast effect was not observed for all three desserts being studied. The assessment was modulated by the interaction between the plate and the type of dessert, as well as the time of day. Similarly, recent results by Kpossa and Lick [46] showed a non-significant effect of plate color (white vs. black) and macaron colors (green, pink, light brown, dark brown, yellow, and off-white) on the hedonic response and purchase intent by consumers. There was no difference in the hedonic value before and after tasting. 

The slightly lower liking of dessert served on a red plate may stem from the fact that red plateware can trigger food avoidance motivation through its association with danger and ‘stop’. Interestingly, these properties of the color red only occur under conditions in which the color has no specific meaning attached (e.g., cola drink) [11]. The liking of food served from red receptacles may be altered by playing music during consumption. Although Cho et al. [47] found that consumers liked cookies more when served on red plates while a high-arousal piece of music was played, a black plate for dessert elevated both liking and price perception. The color black in product design has both positive and negative connotations: it is associated with sophistication, glamour, and technology, but also with fatality, poison, dirt, and toxicity [48].

In a study by Berčík et al. [29], consumers were willing to pay EUR 4.4 for a waffle served on a black stone plate, compared to only EUR 3.5 when served on a white plate. The use of paper and disposable food boxes lowered consumer willingness to pay up to EUR 3.0 for the dish. Other studies highlighted the importance of plating neatness [49], an artistically inspired manner of presentation, and center-oriented plating [23,50]. However, unexpected results were revealed by Zhang et al. [51], where chocolate served on expressive aesthetic plates with low beauty scores was perceived as being more expensive than when it was served on those with high beauty scores. Zhao et al. [14] reported that consumers would pay less for noodles served on blue plates than on red or white plates.

Our study showed a non-significant effect of plate color on energy value perception. Most of the available research focuses on the influence of plate color on food intake. Results from Akyol et al. [52] demonstrated that plate color affected the average total energy intake; a larger amount of pasta with tomato sauce was eaten on red and black plates than on white plates. Rabiei and Nazari [22] demonstrated that the expected satiety level from 200 g of cooked rice eaten from a medium red plate was significantly lower than when eaten from medium and large white plates. The mean values for small white plates were significantly lower than for large white plates only in a group of girls. Reutner et al. [53] have proven that the perceived healthiness of a dish moderates the effect of the color red on consumption. Compared to healthy food, red had a stronger impact on the volume of unhealthy food and the choice of unhealthy meal items. 

**Figure 2:**
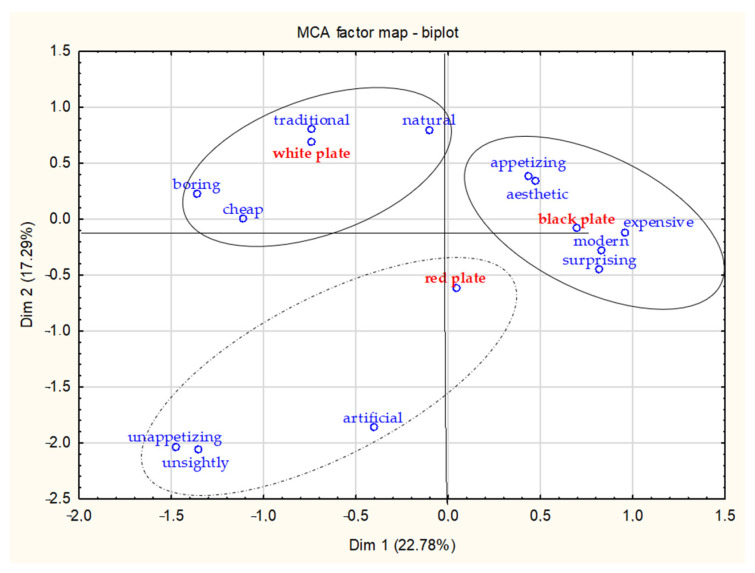
Multiple correspondence analysis of CATA responses on colorful plates associations. **red color**—products; **blue color**—attributes.

The color of the plate significantly modulated sensory and hedonic impressions of dessert given by a group of respondents (Table 5, Figure 2). The use of colored plates resulted in a slightly higher number of affective impressions toward the dish. A bright dessert served on a white plate was perceived by more consumers as traditional (63.9% of respondents), natural (42%), and boring (34.7%). Black plates used to serve dessert aroused other kinds of associations in consumers, who perceived the dish as modern (64.8% of respondents), appetizing (64.7%), and aesthetic (69.6%). Similar associations were found in the case of the red plate. However, the red color evoked more feelings than in other cases, such as being artificial, unsightly, and unappetizing. 

In the study by Jeesan and Seo [13], rice color affected the hedonic response of consumers. The white rice sample was more associated with “good”, “pleased”, “satisfied”, and “pleasant” emotions, while the green color sample was more related to “adventurous” and “wild” emotions. In a study by Chen et al. [54] on the effect of plate color on appetite and promoting enjoyment in Chinese dining, it was shown that white, gold, and black plates were perceived as the most joyful, arousing dining-related emotions and encouraging appetite.

Similarly to our study, participants in a study by Zhao et al. [14] rated noodles served on a white plate as looking the most familiar. The least familiar-looking pasta was served on the blue plate. Food images with a stronger contribution from the red color channel from the Food-pics database were rated as more arousing [10]. In our study, serving dessert on red plates had a negative connotation.

The appetizing nature of a dish was most related to its aesthetics (rho = 0.480, *p* ≤ 0.05), while the modernity of the plates was most related to being surprising (rho = 0.389, *p* ≤ 0.05) and expensive (rho = 0.396, *p* ≤ 0.05). On the other hand, the unappetizing nature of a dish was most related to being unsightly (rho = 0.444, *p* ≤ 0.05) and artificial (rho = 0.371, *p* ≤ 0.05).

Similar results were obtained by other authors. The results of Gunaratne et al. [55] on chocolate packaging revealed that familiar packaging was positively correlated to liking (r = 0.88), while novel packaging did not show any correlation. The findings of Quach et al. [56] demonstrated that neatness enhances the likelihood of a purchase when combined with modern positioning, but non-neat presentation increases the inclination to buy a traditional product due to temporal fit.

### 3.4. Effect of the Food Neophobia Level and Other Socio-Demographic Features on Dessert Perception

The food neophobia level had a significant effect on consumer perceptions of dessert (Table 6). The higher the food neophobia level, the lower the appearance score (rho = −0.059–−0.093, *p* ≤ 0.05) and menu price (rho = −0.053–0.049, *p* ≤ 0.05), while the higher the perceived portion size (rho = 0.081–0.094, *p* ≤ 0.05) and energy value (rho = 0.142–0.144, *p* ≤ 0.05) of the dessert. In the case of colorful plates, the most neophobic subjects perceived the energy value of a dessert as significantly higher than the most neophiliac consumers. The most neophobic subjects scored lower on the appearance of dessert served on white and black plates than others, which was not the case with dessert served on a red receptacle. Consumer innovativeness only affected the perceived energy value and the menu price of the dessert. The more innovative the consumers, the lower the perceived energy value (rho = −0.092, *p* ≤ 0.05) and the higher the expected price of the dish (rho = 0.070, *p* ≤ 0.05). 

The effect of plate size was less affected by FNL. Although appearance was higher in the most neophiliac group compared to the most neophobic, the exaggerated energy value perception (+4.1–8.7%) was stated in the most neophobic consumer group only in the case of the smallest plate. Based on this study, the FNL seems to have an even smaller influence on dish shape perception.

In this study, FNS influenced the liking of black-plate and white-plate desserts, but not of red ones. Annette and Stafford [42] revealed that the degree of snack liking was influenced by receptacle color in picky eaters but not in non-picky eaters. The snack was least desirable when served in the red bowl. These authors provided early-stage evidence that food perception in picky eaters depends on serving receptacle color and may be a simple intervention for those with a restricted dietary repertoire. On the other hand, data from Jaeger et al. [57] suggest that individuals with high levels of food neophobia like food less than those with lower levels of food neophobia, irrespective of their familiarity. Specifically, food neophobes rated lower on the degree of liking for nearly half of food and beverages, including everyday consumables. 

## 4. Limitations

We are aware that our research may have two limitations. The first is the use of a non-representative sample of participants, so data should not be related to the general population of Poles. The second is the use of overhead photos of plates instead of presenting them in reality. These limitations are evidence of the difficulty of collecting data on a large sample of subjects.

## 5. Conclusions

This study explored the effect of various food plate features on dish perception in terms of visual attractiveness, portion size, energy value, and expected price. The results based on the computer-assisted web-based interviewing approach method in a group of 1005 Polish residents revealed that plate size (Ø = 24, 27, 31cm), shape (round, rectangular, square), and color (white, red, black) had a significant effect on a given dish’s perception.

Our findings demonstrated that when plate size increased, the dish’s perceived appearance (*p* ≤ 0.001), perceived portion size (*p* ≤ 0.001), and perceived energy value (*p* ≤ 0.01) were lowered. Remarkably, the portion size and calorific value of the dessert served on the biggest plate were the closest to the actual value. Plate shape only affected the perceived appearance. Round plates seem to be seen as the standard, although angular-shaped plates had supporters and critics in the study group. Plate color had a major effect on the dish’s perceived appearance (*p* ≤ 0.001) and its value in PLN (*p* ≤ 0.001), but it also elicited a greater number of sensory–hedonic reactions. Desserts served on colorful plates were perceived as more expensive than those served on white vessels, as a significant number of consumers perceived bright cream served on white plates as traditional, natural, and boring. The majority of customers perceived dessert on black plates as modern, appetizing, and aesthetic. The level of consumer neophobia influenced the perceived appearance, portion size, and energy value of desserts. Higher food neophobia corresponded to lower perceived appearance and perceived menu price but higher portion size and energy value appraisal.

Although a significant effect of plate features on the examined aspects was found, these data need to be interpreted with caution, given the practical magnitude of the changes involved.

## Figures and Tables

**Table 1:** Characteristics of respondents.

Feature	Group	Respondents	
		Number (n)	Percentage (%)
Total	-	1005	100.0
Gender	Women Men	770 235	76.6 23.4
Age	18–25 years old 26–40 years old 41–55 years old >56 years old	667 135 161 42	66.4 13.4 16.0 4.2
Education	Vocational or primary school Secondary school Higher education (university)	152 485 384	14.9 47.5 37.6
Dwelling Place	City Village	712 131	70.8 29.2
Financial Situation in Own Opinion	Very good Good Not good, not bad bad	151 535 291 28	15.0 53.2 29.0 2.8
Food Neophobia Level	The most neophiliac (10.0–21.4) The most neutral (21.4–41.8) The most neophobic (41.8–70.0)	479 482 44	47.7 48.0 4.4
Domain-Specific Innovativeness Level	Adapters (6–21) Neutrals (22–26) Innovators (27–42)	448 306 251	44.6 30.4 25.0

**Table 2:** Effect of plate size on the dessert perception in the group of respondents (n = 1005).

Evaluated Features	Mean Values + SE			
	24 cm	27 cm	31 cm	*p*
appearance (1–11 c.u.*)	7.60 ^c^ ± 0.06	7.20 ^b^ ± 0.06	6.60 ^a^ ± 0.07	0.000
portion size (g)	217.07 ^b^ ± 1.43	213.31 ^b^ ± 1.44	205.96 ^a^ ± 1.52	0.000
energy value (kcal)	987.43 ^b^ ± 7.12	979.43 ^b^ ± 7.35	958.47 ^a^ ± 7.38	0.008
menu price (PLN)	14.07 ^a^ ± 0.08	13.94 ^a^ ± 0.08	13.96 ^a^ ± 0.09	0.595

^a, b, c^—the mean values marked by different letters in rows differ significantly. Authentic portion size was 200 g, energy value was 950 kcal, and price was set at PLN 12 (Euro 2.7). *—conventional unit.

**Table 3:** Effect of plate shape on the dessert perception by respondents (n = 1005).

Evaluated Features	Mean Values + SE			
	Round Plate	Square Plate	Rectangular Plate	*p*
appearance (1–11 c.u.*)	7.80 ^b^ ± 0.07	7.50 ^a^ ± 0.13	8.00 ^b^ ± 0.15	0.049
portion size (g)	210.09 ^a^ ± 1.70	213.51 ^a^ ± 3.24	211.57 ^a^ ± 4.41	0.638
energy value (kcal)	956.98 ^a^ ± 8.43	978.30 ^a^ ± 16.92	958.47 ^a^ ± 22.29	0.528
menu price (PLN)	14.17 ^a^ ± 0.10	14.18 ^a^ ± 0.19	14.26 ^a^ ± 0.23	0.940

^a, b^—the mean values marked by different letters in rows differ significantly. Authentic portion size was 200 g, energy value was 950 kcal, and menu price was set at PLN 12 (Euro 2.7). *—conventional unit.

**Table 4:** Effect of plate color on the dessert perception by respondents (n = 1005).

Evaluated Features	Mean Values + SE			
	White Plate	Black Plate	Red Plate	*p*
appearance (1–11 c.u.)	7.10 ^a^ ± 0.07	8.00 ^b^ ± 0.06	6.80 ^a^ ± 0.08	0.000
portion size(g)	213.74 ^a^ ± 1.45	217.62 ^a^ ± 1.47	214.53 ^a^ ± 1.43	0.117
energy value(kcal)	981.63 ^a^ ± 7.32	992.88 ^a^ ± 7.58	988.96 ^a^ ± 7.41	0.502
menu price (PLN)	13.93 ^a^ ± 0.09	14.72 ^c^ ± 0.09	14.22 ^b^ ± 0.09	0.000

^a, b, c^—the mean values marked by different letters in rows differ significantly. Authentic portion size was 200 g, energy value was 950 kcal, and menu price was set at PLN 12 (Euro 2.7).

**Table 5:** Sensory and hedonic impressions toward dessert served on colorful plates by respondents (n = 1005).

Attributes	Frequency of Citations		
	White Plate	Black Plate	Red Plate
surprising	0.103 ^a^	0.405 ^b^	0.399 ^b^
boring	0.347 ^c^	0.053 ^a^	0.087 ^b^
traditional	0.639 ^c^	0.151 ^a^	0.198 ^b^
modern	0.160 ^a^	0.648 ^c^	0.445 ^b^
appetizing	0.516 ^a^	0.647 ^b^	0.522 ^a^
unappetizing	0.060 ^b^	0.037 ^a^	0.114 ^c^
cheap	0.259 ^c^	0.062 ^a^	0.017 ^b^
expensive	0.122 ^a^	0.438 ^c^	0.234 ^b^
aesthetic	0.500 ^a^	0.696 ^b^	0.534 ^a^
unsightly	0.047 ^a^	0.032 ^a^	0.119 ^b^
natural	0.420 ^b^	0.233 ^a^	0.228 ^a^
artificial	0.024 ^a^	0.074 ^b^	0.238 ^c^
Total number of citations	3213	3491	3305

^a, b, c^—mean values marked by different letters in rows differ significantly at *p* ≤ 0.0001. Multiple pairwise comparisons using the McNemar (Bonferroni) was applied.

**Table 6:** Effect of food neophobia level on the dessert perception by respondents (n = 1005).

Feature	Food Neophobia Level	Appearance	Portion Size	Energy Value	Dessert Price
red plate	Neophiliac	6.8 ^a^ ± 0.2	211,6 ^a^ ± 3.26	948.4 ^a^ ± 17.0	14.6 ^a^ ± 0.2
	Neutral	6.8 ^a^ ± 0.1	215.7 ^a^ ± 1.72	990.4 ^a,b^ ± 8.9	14.2 ^a^ ± 0.1
	Neophobic	6.6 ^a^ ± 0.2	212.6 ^a^ ± 4.11	1030.7 ^b^ ± 20.5	13.9 ^a^ ± 0.3
black plate	Neophiliac	8.3 ^b^ ± 0.1	213.1 ^a^ ± 3.24	948.4 ^a^ ± 17.5	15.1 ^a^ ± 0.2
	Neutral	8.0 ^a,b^ ± 0.1	219.2 ^a^ ± 1.76	997.9 ^a^ ± 17.0	14.7 ^a^ ± 0.1
	Neophobic	7.4 ^a^ ± 0.2	215.7 ^a^ ± 4.37	1024.2 ^b^ ± 22.2	14.3 ^a^ ± 0.2
white plate	Neophiliac	7.3 ^b^ ± 0.1	210.1 ^a^ ± 3.11	947.8 ^a^ ± 17.0	14.3 ^a^ ± 0.2
	Neutral	7.1 ^a,b^ ± 0.1	215.1 ^a^ ± 1.76	983.5 ^a,b^ ± 8.7	13.9 ^a^ ± 0.1
	Neophobic	6.6 ^a^ ± 0.2	212.1 ^a^ ± 4.18	1014.4 ^b^ ± 20.9	13.6 ^a^ ± 0.25
	Neophiliac	7.9 ^b^ ± 0.1	213.8 ^a^ ± 3.2	953.3 ^a^ ± 16.4	14.4 ^a^ ± 0.2
24 cm	Neutral	7.6 ^a,b^ ± 0.1	218.4 ^a^ ± 1.7	985.7 ^a^ ± 8.5	14.1 ^a^ ± 0.1
	Neophobic	7.2 ^b^± 0.2	215.0 ^a^ ± 4.27	1036 ^b^ ± 19.8	13.7 ^a^ ± 0.25
	Neophiliac	7.4 ^b^ ± 0.1	211.0 ^a^ ± 3.16	947.8 ^a^ ± 17.2	14.2 ^a^ ± 0.2
27 cm	Neutral	7.1 ^a,b^ ± 0.1	214.6 ^a^ ± 1.73	981.5 ^a^ ± 8.7	13.9 ^a^ ± 0.1
	Neophobic	6.8 ^a^ ± 0.2	210.1 ^a^ ± 4.23	1007.7 ^a^ ± 20.8	13.7 ^a^ ± 0.2
31 cm	Neophiliac	6.8 ^a^ ± 0.2	203.3 ^a^ ± 3.44	926.8 ^a^ ± 17.6	14.3 ^a^ ± 0.2
	Neutral	6.7 ^a^ ± 0.1	206.8 ^a^ ± 1.84	960.7 ^a^ ± 8.8	13.9 ^a^ ± 0.11
	Neophobic	6.3 ^a^ ± 0.2	205.3 ^a^ ± 4.24	985.8 ^a^ ± 20.6	13.7 ^a^ ± 0.24
round plate (31 cm)	Neophiliac	8.1 ^a^ ± 0.1	211.5 ^a^ ± 3.4	923.0 ^a^ ± 17.9	14.4 ^b^ ± 0.2
	Neutral	7.8 ^a^ ± 0.1	211.5 ^a^ ± 2.1	960.1 ^a^ ± 10.1	14.3 ^a,b^ ± 0.1
	Neophobic	7.4 ^a^ ± 0.2	202.3 ^a^ ± 5.1	986.7 ^a^ ± 25.6	13.5 ^a^ ± 0.3
square plate	Neophiliac	8.1 ^a^ ± 0.2	211.9 ^a^ ± 5.2	947.4 ^a^ ± 26.7	14.0 ^a^ ± 0.3
	Neutral	7.5 ^a^ ± 0.2	215.3 ^a.b^ ± 3.9	990.4 ^a^ ± 19.6	14.2 ^a^ ± 0.2
	Neophobic	7.1 ^a^ ± 0.3	221.8 ^b^ ± 8.5	1022.0 ^a^ ± 40.9	13.3 ^a^ ± 0.6
rectangular plate	Neophiliac	7.9 ^a^ ± 0.4	212.9 ^a^ ± 9.7	966.8 ^a^ ± 51.0	14.8 ^a^ ± 0.6
	Neutral	8.1 ^a^ ± 0.2	211.9 ^a^ ± 5.2	947.4 ^a^ ± 26.7	14.0 ^a^ ± 0.3
	Neophobic	7.6 ^a^ ± 0.7	207.3 ^a^ ± 16	1019.1 ^a^ ± 69.1	14.9 ^a^ ± 0.8

^a, b^—mean values marked by different letters in rows, differ significantly at *p* ≤ 0.05.

## Data Availability

The data presented in this study are in Polish and are available on request from the first author.

## Supplementary Materials

The following supporting information can be downloaded at: https://www.mdpi.com/article/10.3390/foods13132063/s1, Table S1: Questionnaire; Figure S2: Collage of photos used in the study.
