# Supplementary material for: Effect of Shape, Size, and Color of the Food Plate on Consumer Perception of Energy Value, Portion Size, Attractiveness, and Expected Price of Dessert
Source: Foods. 2024 Jun 28;13(13):2063. doi: 10.3390/foods13132063 (PMC11241694; doi:10.3390/foods13132063)
Supplement: Supplementary file 1 [file foods-13-02063-s001.zip › foods-3038691-supplementary.pdf]

The effect of the dessert presentation on its perception by consumers

You are taking part in a survey focused on the evaluation of the dessert plating manner (vanilla-cream mousse on a crispy chocolate crust with fruit) on the consumers' perception. Begin the survey by completing the first part of the survey, and then proceed to the next section

Consent: I have read and understand the above consent form. I certify that I am 18 years old or older. By clicking the "Next" button to enter the survey, I indicate my willingness to voluntarily take part in this study.

1. Do you use catering services? ****Select only one answer.*

- ☐ Yes
- ☐ No

2. Which type of gastronomic unit do you choose most often? ****Select only one answer.*

- ☐ restaurant
- ☐ bar
- ☐ fast-food
- ☐ canteen
- ☐ café
- ☐ street food

3. Do you pay attention to the way the dishes are served? ****Select only one answer.*

- ☐ Yes
- ☐ No
- ☐ Not sure

4. What elements do you pay attention to after receiving the dish? (Please mark an X in the appropriate boxes)

- ☐ Overall appearance
- ☐ The plate/dish in which the food is served
- ☐ Size
- ☐ Decorative elements/edible decorations
- ☐ The way it is arranged on the plate
- ☐ Smell
- ☐ Taste
- ☐ Consistency

5. Are you interested in cooking? *Select only one answer.

- ☐ Yes
- ☐ No
- ☐ Not sure

6. If yes, how: *Check all appropriate answers.

- ☐ I watch culinary TV programs
- ☐ I follow culinary blogs
- ☐ I go on tastings in fine dining restaurants
- ☐ I participate in culinary workshops
- ☐ I buy culinary publications (books, press, etc.)

7. Have you ever heard the term "plating"? *Select only one answer.

- ☐ Yes
- ☐ No
- ☐ Not sure

8. To what extent do you agree with the following statements? please mark X in the appropriate box*

Table S1. Questionnaire

Statements	strongly disagree	disagree	more or less disagree	undecided	more or less agree	agree	strongly agree
	1	2	3	4	5	6	7
I am constantly sampling new and different cuisines.							
I do not trust new foods.							
If I do not know what is in a food, I will not try it.							
I like foods from different countries.							
Food that I have not eaten before, look too weird to eat.							
At dinner parties I will try a new food.							
I am afraid to eat things I have never had before.							
I am very particular about the foods I will eat.							
I will eat almost anything.							
I like to try restaurants with new cuisines.							
I buy new foods before other people do							
In general, I am among the first in my circle of friends to buy new foods							
Compared to my friends I buy more new foods							
Even though new foods are available in the store, I do not buy them							
In general, I am the last in my circle of friends to know the trademarks of new foods.							
I will not buy new foods, if I have not tasted them yet.							

SECTION ONE

A different way of presenting the cream mousse is presented below. The photos present the dessert from different perspectives to make it easier to answer later in the survey. Please specify the attractiveness of the overall appearance, portion size, energy value, and the maximum price you would be able to pay for the dessert.

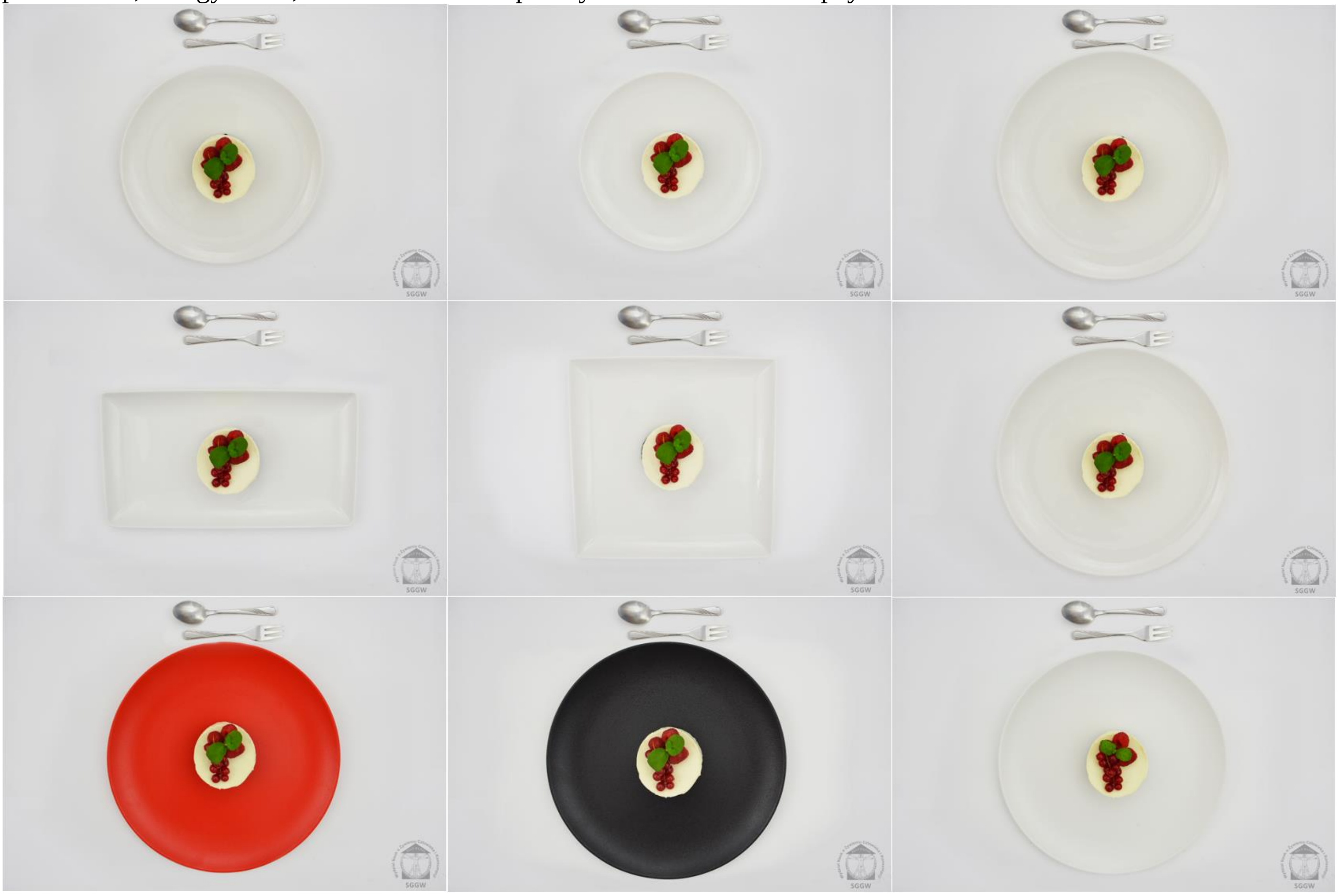

Figure S2. Collage of photos used in the study

Please rate the visual appeal of the presented dessert

dislike ○ ○ ○ ○ ○ ○ ○ ○ ○ ○ ○ like very much

Please estimate the portion size of the presented dessert

100 g ○ ○ ○ ○ ○ ○ ○ ○ ○ ○ ○ 300 g

Please estimate the energy value of the presented dessert

475 kcal ○ ○ ○ ○ ○ ○ ○ ○ ○ ○ ○ 1425 kcal

Please specify the price, that you would be able to pay for the presented dessert

6 zł ○ ○ ○ ○ ○ ○ ○ ○ ○ ○ ○ 18 zł

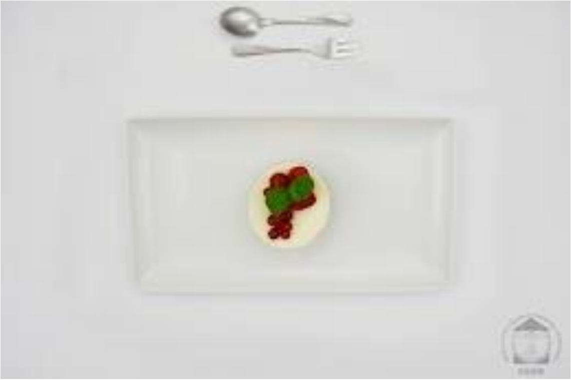

Please rate the visual appeal of the presented dessert

dislike ○ ○ ○ ○ ○ ○ ○ ○ ○ ○ ○ like very much

Please estimate the portion size of the presented dessert

100 g ○ ○ ○ ○ ○ ○ ○ ○ ○ ○ ○ 300 g

Please estimate the energy value of the presented dessert

475 kcal ○ ○ ○ ○ ○ ○ ○ ○ ○ ○ ○ 1425 kcal

Please specify the price, that you would be able to pay for the presented dessert

6 zł ○ ○ ○ ○ ○ ○ ○ ○ ○ ○ ○ 18 zł

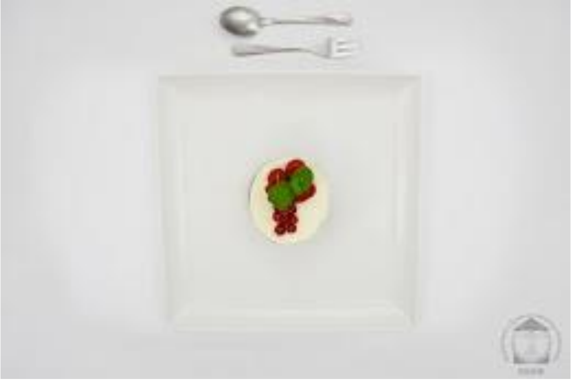

Please rate the visual appeal of the presented dessert

dislike ○ ○ ○ ○ ○ ○ ○ ○ ○ ○ ○ like very much

Please estimate the portion size of the presented dessert

100 g ○ ○ ○ ○ ○ ○ ○ ○ ○ ○ ○ 300 g

Please estimate the energy value of the presented dessert

475 kcal ○ ○ ○ ○ ○ ○ ○ ○ ○ ○ ○ 1425 kcal

Please specify the price, that you would be able to pay for the presented dessert

6 zł ○ ○ ○ ○ ○ ○ ○ ○ ○ ○ ○ 18 zł

SECTION TWO

Please look at the dessert and its appearance on the presented plate and then evaluate the elements given below (attractiveness of the overall appearance, portion size, energy value, and the maximum price you would be able to pay for the dessert).

1) Plate 1

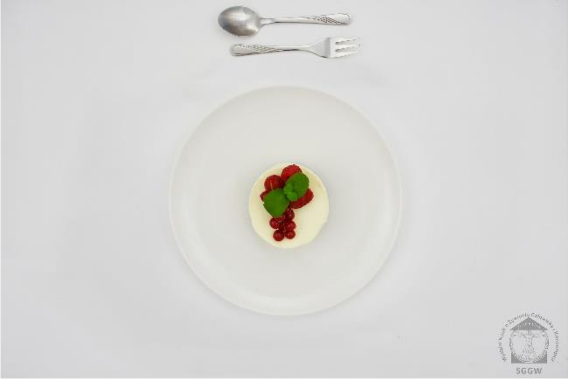

Please rate the visual appeal of the presented dessert

dislike ○ ○ ○ ○ ○ ○ ○ ○ ○ ○ ○ like very much

Please estimate the portion size of the presented dessert

100 g ◯ ◯ ◯ ◯ ◯ ◯ ◯ ◯ ◯ ◯ ◯ 300 g

Please estimate the energy value of the presented dessert

475 kcal ◯ ◯ ◯ ◯ ◯ ◯ ◯ ◯ ◯ ◯ ◯ 1425 kcal

Please specify the price, that you would be able to pay for the presented dessert

6 zł ◯ ◯ ◯ ◯ ◯ ◯ ◯ ◯ ◯ ◯ ◯ 18 zł

2) Plate 2

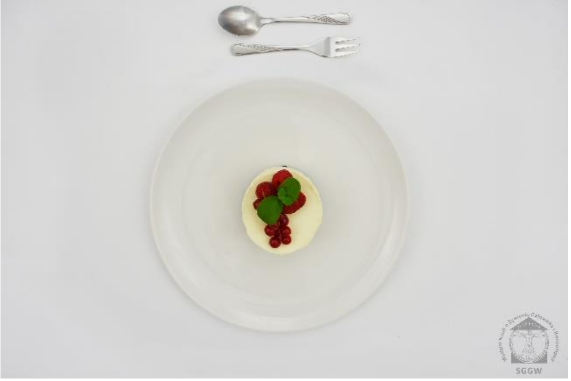

Please rate the visual appeal of the presented dessert

dislike ○ ○ ○ ○ ○ ○ ○ ○ ○ ○ ○ like very much

Please estimate the portion size of the presented dessert

100 g ○ ○ ○ ○ ○ ○ ○ ○ ○ ○ ○ 300 g

Please estimate the energy value of the presented dessert

475 kcal ○ ○ ○ ○ ○ ○ ○ ○ ○ ○ ○ 1425 kcal

Please specify the price, that you would be able to pay for the presented dessert

6 zł ○ ○ ○ ○ ○ ○ ○ ○ ○ ○ ○ 18 zł

3) Plate 3

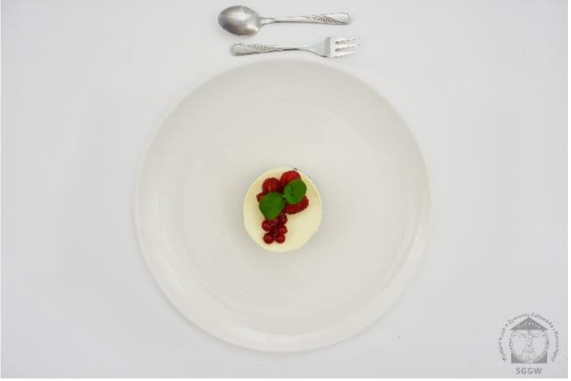

Please rate the visual appeal of the presented dessert

dislike ○ ○ ○ ○ ○ ○ ○ ○ ○ ○ ○ like very much

Please estimate the portion size of the presented dessert

100 g ○ ○ ○ ○ ○ ○ ○ ○ ○ ○ ○ 300 g

Please estimate the energy value of the presented dessert

475 kcal ○ ○ ○ ○ ○ ○ ○ ○ ○ ○ ○ 1425 kcal

Please specify the price, that you would be able to pay for the presented dessert

6 zł ○ ○ ○ ○ ○ ○ ○ ○ ○ ○ ○ 18 zł

SECTION THREE

The last task will be to evaluate the dessert on colorful plates. Please look at the dessert and its appearance on the presented plate and then evaluate the elements given below (attractiveness of the overall appearance, portion size, energy value, and the maximum price you would be able to pay for the dessert).

1. **Red plate**

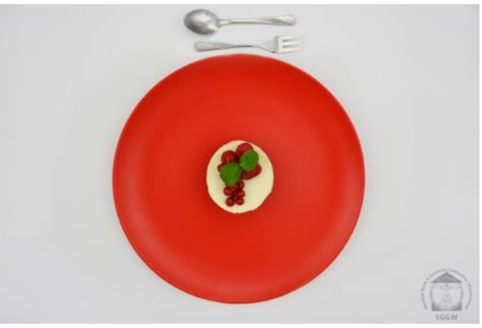

Please rate the visual appeal of the presented dessert

dislike ○ ○ ○ ○ ○ ○ ○ ○ ○ ○ ○ like very much

Please estimate the portion size of the presented dessert

100 g ○ ○ ○ ○ ○ ○ ○ ○ ○ ○ ○ 300 g

Please estimate the energy value of the presented dessert

475 kcal ○ ○ ○ ○ ○ ○ ○ ○ ○ ○ ○ 1425 kcal

Please specify the price, that you would be able to pay for the presented dessert

6 zł ○ ○ ○ ○ ○ ○ ○ ○ ○ ○ ○ 18 zł

Please indicate which you think apply to the dessert you have just seen by placing a cross like this [X] in the box next to it. **There are no right or wrong answers. Please tick as many or as few as you wish.**

	surprising		appetizing		aesthetic
	boring		unappetizing		unsightly
	traditional		cheap		natural
	modern		expensive		artificial

2) Black plate

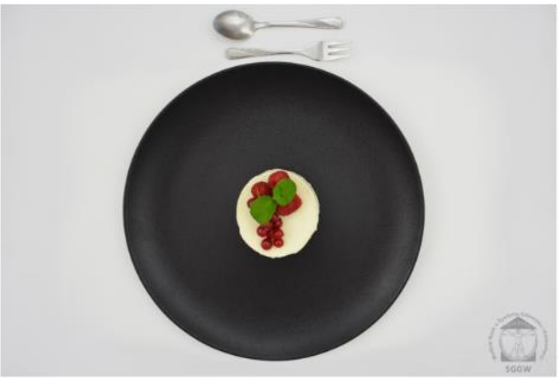

.
Please rate the visual appeal of the presented dessert

dislike ○ ○ ○ ○ ○ ○ ○ ○ ○ ○ ○ like very much

Please estimate the portion size of the presented dessert

100 g ○ ○ ○ ○ ○ ○ ○ ○ ○ ○ ○ 300 g

Please estimate the energy value of the presented dessert

475 kcal ○ ○ ○ ○ ○ ○ ○ ○ ○ ○ ○ 1425 kcal

Please specify the price, that you would be able to pay for the presented dessert

6 zł ○ ○ ○ ○ ○ ○ ○ ○ ○ ○ ○ 18 zł

Please indicate which you think apply to the dessert you have just seen by placing a cross like this [X] in the box next to it. **There are no right or wrong answers. Please tick as many or as few as you wish.**

	surprising		appetizing		aesthetic
	boring		unappetizing		unsightly
	traditional		cheap		natural
	modern		expensive		artificial

3. **White plate**

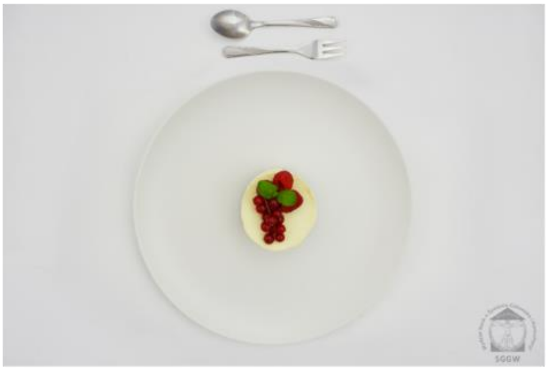

Please rate the visual appeal of the presented dessert

dislike ○ ○ ○ ○ ○ ○ ○ ○ ○ ○ ○ like very much

Please estimate the portion size of the presented dessert

100 g ○ ○ ○ ○ ○ ○ ○ ○ ○ ○ ○ 300 g

Please estimate the energy value of the presented dessert

475 kcal ○ ○ ○ ○ ○ ○ ○ ○ ○ ○ ○ 1425 kcal

Please specify the price, that you would be able to pay for the presented dessert

6 zł ○ ○ ○ ○ ○ ○ ○ ○ ○ ○ ○ 18 zł

Please indicate which you think apply to the dessert you have just seen by placing a cross like this [X] in the box next to it. **There are no right or wrong answers. Please tick as many or as few as you wish.**

	surprising		appetizing		aesthetic
	boring		unappetizing		unsightly
	traditional		cheap		natural
	modern		expensive		artificial

Characteristics of respondent

Finally, please provide sociodemographic information.

1. **Grender** *Select only one answer.
   - ☐ women
   - ☐ men
2. **Age** *Select only one answer.
   - ☐ 18–25 years old
   - ☐ 26–40 years old
   - ☐ 41–55 years old
   - ☐ >56 years old
3. **Dwelling place** *Select only one answer.
   - ☐ city
   - ☐ village
4. **How do you assess your financial situation** *Select only one answer
   - ☐ very good (I have money left at the end of the month for a few luxuries or to add to my savings)
   - ☐ good (I get by, but there's not a lot left by the time the basics are taken care of)
   - ☐ not good not bad (I'm making ends meet, but only just)
   - ☐ bad (I'm in danger of falling behind with bills or loan repayments)
